# Q&A: Scaling up delivery of mental health treatments in low and middle income countries: interviews with Retha Arjadi and Vikram Patel

**DOI:** 10.1186/s12916-018-1209-1

**Published:** 2018-11-19

**Authors:** Retha Arjadi, Vikram Patel

**Affiliations:** 1 Faculty of Psychology, Atma Jaya Catholic University of Indonesia, Jakarta, Indonesia; 2 Department of Clinical Psychology and Experimental Psychopathology, University of Groningen, Groningen, Netherlands; 3 Department of Global Health and Social Medicine, Harvard Medical School, Boston, Massachusetts USA

## Abstract

In this Q&A, we talk with Retha Arjadi and Vikram Patel about using new technologies and lay-counselor support for scalable delivery of mental health treatment in low and middle-income countries.

## Introduction

Retha Arjadi is a clinical psychologist in Jakarta, Indonesia and faculty of psychology at Atma Jaya Catholic University of Indonesia. Her work focuses on using alternatives to traditional face-to-face therapy for delivering treatment to patients with depression in low and middle-income countries. She discusses here recent work using an internet-based platform combined with lay counselor support for delivering treatment for depression in Indonesia [1]. We also talk with Vikram Patel, a leader in global mental health research and professor at Harvard Medical School, Department of Global Health and Social Medicine. He discusses the field of global mental health and efforts to develop scalable methods for delivering treatment, and where he thinks the field is heading. He also comments on Arjadi’s recent work.

### Q&A with Retha Arjadi

#### How did you get interested scalable delivery of mental health treatment in low and middle income countries?

I’ve always been interested in any kind of research on depression and any research on the intervention of depression. As we know, from the World Health Organization, we know that 4.4% of the world's population is suffering from depression and depression is also one of the leading causes of global burden of disease. In low and middle income countries specifically, it is reported around 4.2%, and in Indonesia, where I live and conduct research, it accounts for about 3.4%. It seems lower in terms of percentage in Indonesia, but with 260 million people, it equals more than 8 million people suffering from depression in Indonesia, so I think it’s important to do research like this because we do need some more options to deliver health care in low and middle income countries like Indonesia.

#### What was the inspiration for doing your recent project of internet-based treatment in Indonesia?

So I think I’m going to tell you a little bit of my story before I began my PhD. So I was always thinking of doing research or clinical practice using non face-to-face approaches, and I happened to read one of my professor’s papers on telephonic therapy, and I thought that would be great idea to do some similar research like that in Indonesia as well. And then I discussed it with my professor back then, and we thought that telephonic therapy is one option, but we also know that internet use is increasing rapidly all over the world. I live in Jakarta and I can see that everybody is using a smart phone in their daily routine. Because it seems like that is the direction things are going, we thought that we can utilize the internet to also deliver mental health services and interventions. So I think this is my personal wish, that more people in Indonesia can have more options to mental health care mental health services because it is quite expensive to go to psychologists here because there is no health insurance that covers it. At least most health insurance from private sources and also from the government does not cover for psychological services. Using the internet, I thought that we could reduce the cost, and more people could have access to this kind of intervention.

#### Would you mind walking us through setting up your project for delivering internet-based therapy for depression in Indonesia?

The internet based behavior activation program that we have used is adapted from a Dutch version of the same kind of intervention. It’s a web-based therapy basically and at first I thought I only had to translate everything from Dutch to bahasa Indonesia, the official language of Indonesia. So I had a research assistant who is a Dutch native student who translated the original Dutch-based platform for me from Dutch to English, and then I translated everything from English to bahasa Indonesia. But then when I did that, I realized that wow, this is very contextual to Dutch culture, and we cannot use this for Indonesian people. For example, in the Netherlands it is common for people to walk the dog in the park, because they have so many nice public parks. But that’s not necessarily the case for most people in Indonesia. When I tried to introduce this example to my colleagues in Indonesia they said this is nonsense, you cannot use these kinds of examples for Indonesian people. You need to adapt everything, not just the language but also the cultural context, including the examples you use on the platform. So with the help of clinical psychologists and other stakeholders, including our lay counselors, we tried to adapt everything to make it culturally appropriate. That definitely took more than a year to finish everything, and then we had to put all of the information and example onto the platform for the website. This also required additional hard work because in the Dutch version they use really nice videos of therapists talking to you. But Indonesia happens to not have the best internet connection in the world, so when I did some pilots with some of my colleagues they said it’s not possible to use video because the slow internet causes it to buffer, which might not be good for delivering therapy. So we turned everything from videos into powerpoint slide shows and imbedded them in the websites. We use only illustrations in black and white with low resolution but still readable and visually appropriate. The important information is still on the platform, but can be delivered even with slower internet connections.

#### Lay counselors played an important role in helping to support the internet-based delivery to patients in a scalable way. Can you tell me about the lay counselors, who they are and how you found them?

I recruited them from the internet, by posting a recruitment poster saying that we were looking for people that had a minimum education background of senior high-school, and care about mental health issues and depression. So everyone could apply and we would then select them based on structured interviews to try to get to know what their purpose is by registering themselves to be the lay counselors in this project. And when we recruited the number of lay counselors we needed, we invited them for a two day intensive training, which included role playing and acting like participants using the platform to ensure they understood the platform and how to help participants go through it.

#### Where do you hope to see this research going in the future?

I really hope to see more studies like this done, not just in Indonesia, but other low and middle income countries as well. I am now collaborating with another PhD student from my professor’s research group that will do a similar study in China, for example. And we hope that more evidence will offer more hope that this might provide ideas for recommendations and policy-making to scale up the mental health services in low and middle income countries. So, while I’m thinking more about how to scale this approach in Indonesia, I would also like to help take it more globally because this type of intervention can also be done in other countries with totally different characteristics, as long as we adapt everything to make it culturally appropriate to those countries. So that's how I see this type of research continuing.

Anything else you want our readers to know about the work? I hope that this research can open the door to more research like this, but I know this is only piece of evidence. So we really need support and the willingness of more researchers to pay more attention to this topic. One piece of evidence can open the door to more studies in this area.

### Q&A with Vikram Patel

#### Tell us about your research group’s background in developing scalable methods for mental health treatment

Our research group’s primary focus for more than ten years has been to experiment with the use of available human resources to deliver mental health interventions, primarily psychological and social interventions. We work across the life course from autism in childhood through to dementia in old age. Our definition of available human resources is very much dependent on the condition that we are dealing with and the context. So in the case of autism, this means working directly with parents, and in the case of other conditions, it is often working with lay people and community health workers. More recently over the last few years, we have begun to experiment with and assess the role of technologies in various different forms to improve detection, access to mental health care and the quality of mental health care.

#### Can you tell us a bit about the history of using non-specialists and other scalable methods for delivering mental health treatment?

It is important to acknowledge that the use of non-specialist and community health workers has a rich history in global health, in diverse areas of health care for decades. Global mental health has been applying this proven method of delivery of health care interventions to the specific area of mental health. A second important factor is the use of empirically supported interventions. Whereas community-based non-specialists workers have been used for mental health care for decades, they tended to deliver interventions that were eclectic and poorly defined, which would not have have met the criteria of evidence as we typically define it in public health which is that they are replicable and proven to work in randomized controlled trials. It is the convergence of these two bodies of evidence, empirically supported interventions with the use of community-based workers to deliver them, that I think has been the real advance in the last decade or so. The field must now head next is to address the question of how we scale this approach up in routine healthcare systems. This requires quite a different approach of implementation-oriented strategies to be designed and evaluated, for example, how do we train health workers and how do we ensure quality of the delivery of care in ways that are scalable.

#### What were some initial thoughts you had about Arjadi et al and using the internet to deliver treatment for depression [1]?

I was very enthused by this paper, and I think as the authors have pointed out, it’s the first demonstration of the effectiveness of an internet delivered therapy in the global context. It provides proof of concept about the feasibility and acceptability of such an intervention, because questions have been often raised about the acceptability and feasibility of internet-delivered interventions in settings where the internet is not as widely used as in the west. This adds of course to the evidence from the high-income world and I think this increasingly shows the value of this approach, this delivery approach in taking psychological therapies to scale.

#### Did anything else strike you about Arjadi et al and their results?

What was very interesting in Arjadi et al was, similar to what we see across many depression trials, the dramatic improvements in the control arm of the trial. This finding reminds us, yet again, that these conditions tend to be self-remitting for a significant proportion of people. I think figuring out who is likely to respond or need an intervention is the next big research question in this field, so that we can approach the goal of personalized allocation of treatments. Imagine if we were able to identify three groups of people before commencing an intervention: 1) those who are going to get better regardless of whether or not they receive any clinical intervention, 2) a second group being those who will respond to a specific clinical intervention, and 3) a third group, those who will not respond at all to the clinical intervention and will therefore need to get something much more intensive. Even the choice of a specific intervention poses potential opportunities for personalized allocation. For example, we now have a number of effective interventions, like anti-depressant medicines and a range of different psychological interventions. The problem is that they are usually compared either head to head, or with a control arm, and you usually get the same results with all of these trials now: these interventions are better than an inactive control arm, but when you compare them head to head there’s really very little to separate them out on the basis of aggregated remission rates. But in all of them you can see that the control arm still does quite well even though they are not getting any active intervention, as well as the that a significant proportion of people in the active intervention arm do not respond. So we really need to move to a next generation of research which really tries to be able to predict which individual does not need any intervention, who will respond to what particular kind of intervention, and who will not respond to any of the known interventions.

#### And do you think that type of prediction is compatible with these scalable methods?

Yes, I do. In fact I think it’s a very relevant question for global health, because it seems very inefficient, for example, to deliver any intervention to someone who doesn’t need it. Even if it’s low-cost, it’s still fairly inefficient because it’s never without a cost. So I think that it’s very inefficient to give an intervention to people who don’t need it. But equally it’s not very efficient to give the intervention to people who won’t respond to it. Equally, if there are multiple types of intervention, I would want to allocate these according to the highest likelihood of a favorable response. I think it is a very important research question, not just from an individual standpoint---if I had depression I ought to know whether or not I would respond to a particular intervention or not---but from a public health point of view, there is obviously a very important imperative from an efficiency perspective. So the exciting research agenda is what are the assessments which can predict treatment response and to what extent can these be scaled up in routine care; this is at the heart of the emerging field of precision psychiatry.
